# The effect of parenteral betanin pre-treatment on the inflammatory response and proliferative activity of pulmonary parenchyma after jejunal ischaemia–reperfusion injury

**DOI:** 10.2478/jvetres-2025-0064

**Published:** 2025-11-18

**Authors:** Milan Maretta, Štefan Tóth, Zuzana Fagová, Martin Urda

**Affiliations:** Department of Neurology, Faculty of Medicine, Pavol Jozef Šafárik University, 041 80 Košice, Slovak Republic; Department of Histology and Embryology, Faculty of Medicine, Pavol Jozef Šafárik University, 041 80 Košice, Slovak Republic; Department of Anesthesia and Intensive Care Medicine, Šaca Hospital, Košice, Slovak Republic

**Keywords:** betalain, PMNL infiltration, reflow injury, remote injury, time-course study

## Abstract

**Introduction:**

Intestinal ischaemia–reperfusion (IR) injury has detrimental effects on both local and distant organs. Serious oxidative damage is caused by reperfusion, and betanin, known for its antioxidant properties, may reduce it. The aim of the present study was to test the hypothesis that betanin administration prior to intestinal IR may be protective of the lung parenchyma against damage inflicted by intestinal IR.

**Material and Methods:**

Forty-nine specific pathogen–free Charles River Wistar albino rats were divided into a sham group (without IR), an IR group (60 min of small-intestine ischaemia with 1, 4 and 24 h of reperfusion – group A and three subgroups) and a betanin-pre-treated IR group (intraperitoneal betanin at 50 mg/kg bw, administration 30 min before ischaemia followed by 1, 4 and 24 h of reperfusion – group B and three subgroups). Lung biopsies were screened for histopathological changes and immunohistochemical expression of anti-cyclooxygenase-2 (COX-2) and anti–proliferating cell nuclear antigen (PCNA).

**Results:**

Pre-treatment with betanin significantly reduced cellular COX-2 expression during the early and late reperfusion periods (respective P-values <0.05 and <0.001) compared to the untreated group. Expression of PCNA was significantly upregulated in both injured groups comparted to the sham group. In betanin-pre-treated rats less than half the PCNA expression noted in the untreated rats was present in the late reperfusion period (group A at 24 h *vs* group B at 24 h; P-value < 0.001).

**Conclusion:**

Betanin pre-treatment prior to intestinal IR is indicated to serve as a protective agent against the lung injury mediated by the intestinal injury.

## Introduction

Intestinal ischaemic–reperfusion injury (IRI) arises as a result of acute mesenteric ischaemia and haemorrhagic shock as medical emergencies or as a result of surgical intervention. Various attempts applying different methods have been made in order to minimise the negative impact of intestinal IRI. Antioxidants have been the object of thorough research (17, 30, 33), and given that reperfusion can induce further tissue damage oxidatively, their effects are highly relevant. The impact of intestinal IRI is far more complex than only local tissue damage. Injuries of this kind to the intestine initiate a complicated pathway which can result in damage to distant organs such as the lungs, kidney and liver (34). This pathway is driven by reactive oxygen species (ROS) produced during the reperfusion period and cytokines mediated by systematic inflammatory response (14, 20, 34). Such damage is observed in lung tissue and is characterised by histopathological (*e.g*. perivascular and interstitial oedema, haemorrhage and inflammatory response) and functional changes and increased microvascular permeability (15). To date, the detailed mechanism of intestinal IR–induced acute lung injury has been poorly understood and there has been no successful line of appropriate treatment.

Health conditions with inadequately comprehended mechanisms are frequent areas where phytogenic compounds are investigated for their potential to be treatments. Betalains are natural colour pigments present in red beetroots (*Beta vulgaris* var. *rubra*). Red beet betalains are composed of two main groups: the red-violet betacyanins (*e.g*. betanin and isobetanin) and the yellow betaxanthins. Numerous studies have revealed the beneficial role of betanin (C_24_H_26_N_2_O_13_), ascribing to it anti-inflammatory (2) and cardioprotective activity (16), antidiabetic activity as it controls the activities of liver marker enzymes (8), lipid peroxidation inhibition and strong antioxidant effect (19, 28). Betanin proved its antioxidant abilities in low concentrations (12). The scavenging activity of betanin is based on its potential to provide an electron to ROS, thus stabilising them. *Beta vulgaris*, its source, has been ranked in the top ten vegetables with the most potent antioxidant effect *in vitro* (3). In a model of paraquat-induced lung injury, betanin proved its antioxidant ability by suppressing polymorphonuclear lymphocyte infiltration; myeloperoxidase and malondialdehyde (MDA) levels in lung tissue; and cytokine production, particularly tumour necrosis factor α (TNF-α) and interleukin 1 β (IL-1β) (16). Betanin was demonstrated to suppress ROS and MDA production and inducible NOS expression, and also to increase the antioxidant capacity of cardiac muscle tissue in a model of isoproterenol-induced acute myocardial infarction (32).

This study arose because the mechanisms of mesenteric IR-induced distant organ damage are incompletely elucidated but certainly involve oxidative stress, effective treatments for the damage are lacking, and betanin possesses potent antioxidant properties. It aimed to establish whether parenteral pre-treatment with this compound protects lung parenchyma in an intestinal IRI model. The objective of this study was to delineate the extent of acute inflammatory and reparative responses in pulmonary tissue during the early reperfusion phase following experimental IRI. This study provides a detailed immunohistochemical description of betanin’s antioxidant effect, utilising quantitative and qualitative methods of investigation of the cells responsible for tissue regeneration and the inflammatory response.

## Material and Methods

The experimental and surgical procedure followed a protocol previously described (30). A total of 49 adult male outbred pathogen-free Charles River albino Wistar rats weighing 250–350 g were used. The animals were housed in standard conditions with controlled temperature (21°C) and had access to commercial chow and water *ad libitum*. Animals were fasted for 12 h before surgery but with free access to water. The rats were randomly divided into three groups as follows: 1 – sham control (SC, n = 7), 2 – experimental groups with betanin (red beet extract diluted with dextrin) pre-treatment (in subgroups B1, n = 7; B4, n = 7; and B24, n = 7), and 3 – experimental groups denied betanin pre-treatment (in subroups A1, n = 7; A4, n = 7; and A24, n = 7). The rats in groups SC and A1, A4 and A24 received an intraperitoneal injection of 0.9% saline solution, which for the A groups was 30 min before the start of ischaemia. The rats in groups B1, B4 and B24 also received this injection at this time, but the saline solution also contained betanin at 50 mg/kg body weight. Rats were subjected to total occlusion of the superior mesenteric artery by using an atraumatic vascular clamp through a 60-min ischaemic interval, following which there was a reperfusion period of 1 h (for groups B1 and A1), 4h (B4 and A4) or 24 h (B24 and A24).

### Tissue specimen preparation

Right middle lung lobe (*lobus medius pulmonis dexter*) biopsies were taken as the most representative lung sample. The biopsies were washed with 0.9% cold saline and fixed in 4% paraformaldehyde. Tissues were then embedded in paraplast, cut into 4–5μm thick sections and mounted. After deparaffinisation, the tissue sections were stained for routine histological and immunohistochemical colorimetric and fluorescent microanalysis.

### Histological analysis of the acute lung injury

In order to assess the lung injury, a grading system was used. The tissue under examination was given a mark from 0–4 based on congestion, interstitial oedema, polymorphonuclear infiltration and airspace haemorrhage as follows: 0 – healthy tissue architecture; 1– focal, mild, subtle changes; 2 – multifocal, mild changes; 3 – multifocal, prominent changes; and 4 – extensive, prominent changes. A histopathological assessment (haematoxylin and eosin) was performed in at least five randomly selected microscopic high-power fields from each lung sample.

### Histochemical and immunohistochemical analysis

The population of cells positive for anti–proliferating cell nuclear antigen (PCNA) and anti-cyclooxygenase-2 (COX-2) in 1 mm^2^ of pulmonary parenchyma was detected in 10 different fields by immunohistochemical analysis. Positive cell populations were visualised with diaminobenzidine (DAB, catalogue No. 32750-1G-F; Sigma-Aldrich, part of Merck, Darmstadt, Germany) and counterstained with Mayer’s haematoxylin. The negative control was established by omitting the primary antibodies. Tissue sections were examined and imaged using a BX50 light microscope with an SP350 camera (Olympus, Tokyo, Japan). Tissue samples were evaluated by two blinded and independent histologists. In each sample at least 10 randomly selected visual fields were observed in 400× magnification. All positive cells were counted in each field using cytometric Quick-PHOTO Industrial 2.3 image analyser software (Promicra, Prague, Czech Republic).

The population of cells in proliferation and DNA repair was detected in five different fields by immunohistochemical analysis using anti-PCNA Mouse Monoclonal Antibody (catalogue No. Ms-106-P1; BioLegend, SanDiego, CA, USA) at 1 : 100 titre in 1 mm^2^ of the pulmonary parenchyma. Positive cell populations were visualised with diaminobenzidine and counterstained with Mayer’s haematoxylin. The negative control was established by omitting the primary anti-PCNA antibody. Rabbit polyclonal primary antibody against active COX-2 (anti-COX-2, catalogue No. RB-9072-R7; Thermo Fisher Scientific, Waltham, MA, USA) was applied at 1 : 100 titre, and the sections were incubated with Goat pAb to Rb IgG (immunoglobulin G) H&L (heavy and light chain) secondary antibody, Alexa Fluor 488 (catalogue No. ab150077; Abcam, Cambridge, UK). Sections were stained for immunofluorescence as described above, washed three times in phosphate-buffered saline, cover-slipped in H-1200 VECTASHIELD with DAPI (4′,6-diamidino-2-phenylindole) mounting medium for fluorescence (Vector Laboratories, Burlingame, CA, USA), and examined under a DM2500 fluorescent microscope (Leica Microsystems, Wetzler, Germany) with ultraviolet and fluorescein isothiocyanate filters. Under the fluorescent microscope at 1,000× magnification, the amount of COX-2-positive cells was detected by positive staining of the cytoplasm (active COX-2) and counted in five randomly-selected fields in the pulmonary parenchyma per section. Immunofluorescent density of COX-2 positive cells in the peribronchiolar lung parenchyma was calculated as the number of cells per unit of lung tissue area (mm^2^). The amount of COX-2-positive epithelial cells in epithelial lining of terminal bronchioles was expressed as a percentage (%) of the total number of bronchiolar epithelial cells. The negative control was established by omitting the primary anti-COX2 antibody.

### Statistical evaluation

Statistical analysis was performed using GraphPad In-Stat version 3.01 (GraphPad Software, San Diego, CA, USA). Quantitative results of immunohistochemical analyses were determined using the one-way analysis of variance test with a multiple comparison Tukey–Kramer *post-hoc* test. The results were expressed as mean ± standard error of the mean. Values of P less than 0.05 were considered to be significant.

## Results

**Fig. 1.**
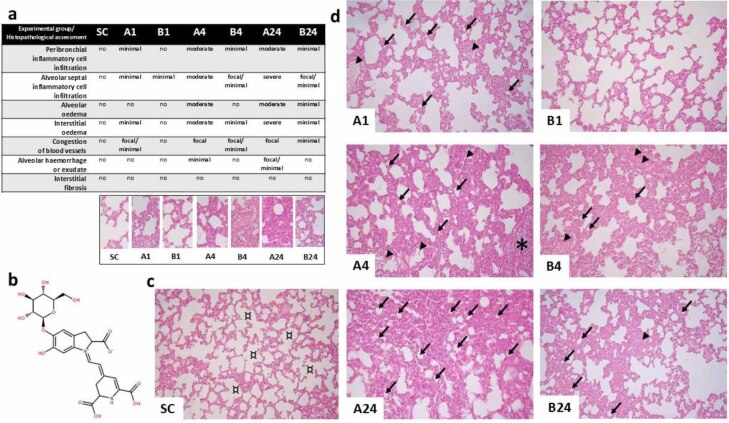
Histopathological findings after mesenteric ischaemia–reperfusion lung injury in albino Wistar rats. (a) Table of histopathological assessment of lung parenchyma lesions for each group. (b) Chemical structure of betanin (C_24_H_26_N_2_O_13_). (c) Fluorescence micrograph of normal lung architecture with no histopathological changes in the sham control (SC) group; ¤ – normal pulmonary alveoli. (d) Fluorescence micrographs showing histopathological changes in the lung parenchyma in each experimental group without (A1, A4 and A24 (numbers denoting reperfusion h)) and with betanin pre-treatment (B1, B4 and B24); arrowhead – congestion of blood vessels; ↙ – prominent alveolar surface reduction with significant interalveolar septa thickening due to interstitial oedema; * – inflammatory cell infiltration. Staining was with haematoxylin and eosin; 400× magnification; scale bar = 200 μm in all micrographs

Routine haematoxylin and eosin staining of lung parenchyma sections from the experimental groups showed that intestinal IR injury resulted in the characteristic features of acute pulmonary injury, whereas lung sections from the SC group showed normal architecture with no histopathological lesions (Fig. 1c). Distinct and severe multifocal histopathological changes in lung parenchyma biopsied after 24 h of reperfusion were found in the group without betanin application. After 24 h of reperfusion in the betanin-pre-treated group, attenuation of histopathological damage was observed (Fig. 1a and 1d).

Lung tissue lesions were present both in interalveolar septa and pulmonary interstitia. Inflammatory cell infiltration, blood vessel congestion and interstitial oedema were observed. These histopathological lesions were mainly detected in the perivascular loose connective tissue (Fig. 1a and 1d) in groups A4 and A24. Histopathological parameters, including inflammatory cell populations, oedema and vascular congestion occurrences were lower in all betanin-pre-treated groups with intestinal ischaemia–reperfusion injury than in untreated groups. Injury in the lung was only more severe in the B4 group than in the B24 group. Presumably, this is a result of the culmination of the inflammatory response and subsequent mitigation.

**Fig. 2.**
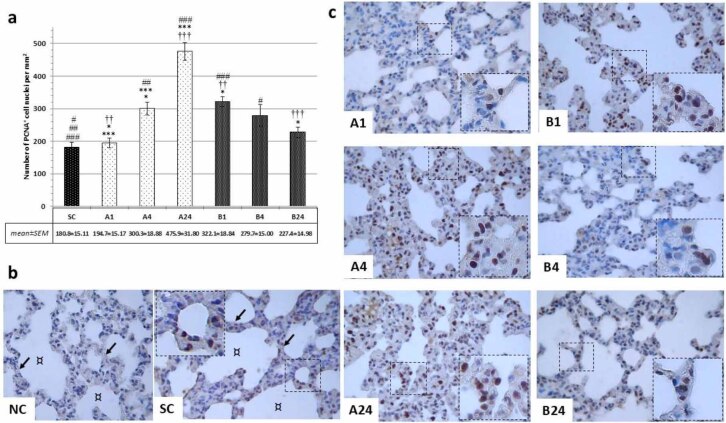
Immunohistochemical findings after mesenteric ischaemia–reperfusion lung injury in albino Wistar rats regarding proliferating cell nuclear antigen (PCNA)-positive cell populations in lung parenchyma. (a) Number of PCNA+ cell nuclei per mm^2^ for each group; # and * – significant difference at P-value < 0.05; ##, ** and †† – significant difference at P-value < 0.01; ###, *** and ††† – significant difference at P-value < 0.001. (b) Brightfield micrographs of pulmonary parenchyma of negative control (NC) without primary antibody application in the betanin-pre-treated group (B) and sham control group (SC); ↙ – interalveolar septa; ¤ – pulmonary alveoli. (c) Brightfield micrographs of the positive cell population in the lung parenchyma in each experimental group without (A1, A4 and A24 (numbers denoting reperfusion h)) and with betanin-pre-treatment (B1, B4 and B24). 1,000× magnification; detail: 1,000×; scale bar = 100 μm in all micrographs

Assessment of PCNA markers noted clear dark-brown–stained nuclei in positive cells. In the SC group a significantly lower number of PCNA-positive cells than in almost all experimental groups was observed (SC *vs* B4, P-value < 0.05; SC *vs* A4, P-value < 0.01; and SC *vs* A24 and B1, P-value < 0.001), only the A1 and B24 groups yielding fewer such cells. In groups denied betanin, gradually more PCNA positivity (A1 *vs* A4, P-value < 0.05; and A1 *vs* A24 and A4 *vs* A24, P-value < 0.001) was noted, with more than a two-fold increase in the late reperfusion period (475.9 ± 31.8 *vs* 180.6 ± 15.11, P-value < 0.001) compared to the control group. In betanin-pre-treated rats an over 50% lower extent of PCNA positivity was noted after 24 h of reperfusion compared to groups without betanin application (227 ± 14.98 (B24) *vs* 475.9 ± 31.8 (A24), P-value < 0.001 (Fig. 2a and 2c).

**Fig. 3.**
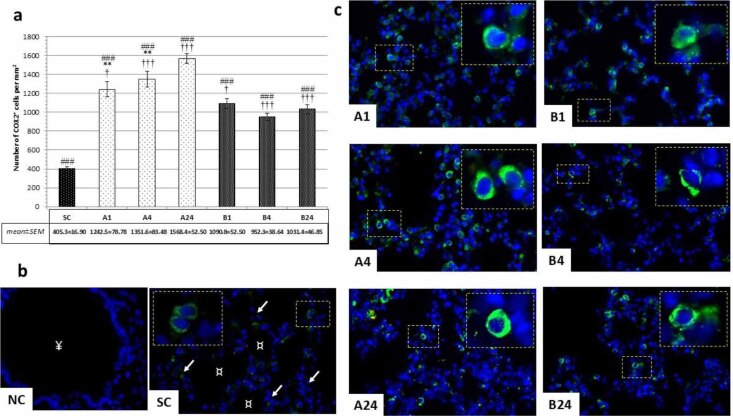
Immunohistochemical findings after mesenteric ischaemia–reperfusion lung injury in albino Wistar rats regarding COX-2+ cell populations in lung parenchyma. (a) Numbers of COX-2^+^ cells per mm^2^ in peribronchiolar lung parenchyma in all groups; † – significant difference at P-value < 0.05; ** – significant difference at P-value < 0.01; ### and ††† – significant difference at P-value < 0.001. (b) Immunofluorescence micrographs of pulmonary parenchyma of negative control (NC) without primary antibody application in the betanin-pre-treated group (B) and sham control group; ↙ – interalveolar septa; ¤ – pulmonary alveoli; ¥ – respiratory bronchiolus. (c) Immunofluorescence micrographs of COX-2^+^ cell populations in peribronchiolar lung parenchyma around bronchioles in each experimental group without (A1, A4 and A24 (numbers denoting reperfusion h)) and with betanin pretreatment (B1, B4 and B24). 600× magnification; detail: 1,000×; scale bar = 50 μm in all micrographs

Immunofluorescence microscopic examination revealed distinct cytoplasmic COX-2-positivity within reactive lung tissue cells. From the results it was clear that intestinal IRI altered COX-2 expression regardless of pre-treatment having been given. In both experimental groups (A and B), significantly higher COX-2-positivity was observed than in group SC (P-value < 0.001; Fig. 3a and 3b). A gradual increase of cytoplasmic COX-2 immunopositivity was noted in group A with almost an almost four-fold increase in the late reperfusion period from 405.3 ± 16.9 to 1568.5 ± 52.5 (P-value < 0.01; Fig. 3a and 3c). In group B, significantly lower cytoplasmic COX-2 positivity was found than in group A in all reperfusion periods (B1 *vs* A1, P-value < 0.05; B4 *vs* A4 and B24 *vs* A24, P-value < 0.001; Fig. 3a and 3c). No significant difference was observed within group B between the three reperfusion periods.

**Fig. 4.**
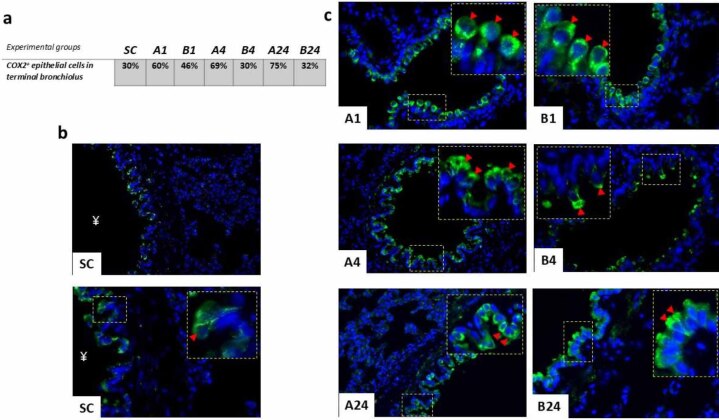
Immunohistochemical findings after mesenteric ischaemia–reperfusion lung injury in albino Wistar rats regarding COX-2^+^ cell populations in the epithelial lining of terminal bronchioles of lung parenchyma. (a) Table of % of COX-2^+^ epithelial cell populations for each group. (b) Immunofluorescence micrograph of the pulmonary parenchyma of the sham control (SC) group with no ischaemia–reperfusion injury; ¥ – lumen of terminal bronchiolus; arrowhead – detail of COX-2^+^ reactivity of club cell cytoplasm. 400× and 600×; scale bar = 50 μm. (c) Immunofluorescence micrographs of positive epithelial cell populations in the terminal bronchioles in each experimental group without (A1, A4 and A24 (numbers denoting reperfusion h)) and with betanin pre-treatment (B1, B4 and B24); arrowhead – detail of COX-2^+^ reactivity of club cell cytoplasm. 600× magnification; detail: 1,000×; scale bar = 50 μm

Besides in lung parenchyma tissue, strong and clear COX-2-positivity was also registered within the epithelial cell lining of terminal bronchioles. Immunofluorescent positivity was clearly and strongly cytoplasmic; histologically, most of those cells were probably non-ciliated secretory club cells (CCs) (Fig. 4b). The IRI caused a gradual increase in COX-2-positive bronchiolar epithelial cells, reaching its peak after 24 h of reperfusion (75%) (Fig. 4a). The highest COX-2 positivity of any group was measured in the B1 group (46%) and the equal lowest in the B4 group (30%), which had the same positivity as the SC group.

## Discussion

It is well known from numerous studies that an IRI in the small intestine has a destructive effect on local tissue, leading to injury, necrosis, accelerated apoptosis and local inflammatory changes. However, reperfusion also severely affects distant organs. The kidney, liver and other organs are in danger from the systematic inflammatory response driven by circulating bacteria from the small intestine (27, 34). Lung injury is one of the most evident complications, manifested by neutrophil accumulation, increased microvascular permeability, perivascular and interstitial oedema, and possibly pulmonary oedema and endothelial cell death (11, 21). In our current study, we observed that intestinal IRI severely affected lung tissue at the histopathological level, exacerbating inflammation and disrupting normal cell turnover in the reperfusion period.

The lung is the second most affected organ after the small intestine in case of intestinal IRI. The mechanism of lung injury involves several steps, including the translocation of bacteria and endotoxins into systemic circulation and the activation of an inflammatory response mediated by pro-inflammatory cytokines (TNF-α and IL-1), and the oxidative stress and cellular and humoral immune system response induces additional organ damage (26, 31). Beside cytokines and other pro-inflammatory mediators and enzymes involved in the production of ROS, COX-2 was suggested to play a pivotal role in the resolution of lung injury. Under physiological conditions, COX-2 expression in the lung is mostly found in epithelial and endothelial cells, type II pneumocytes, macrophages and resident inflammatory cells (35). Acute lung injury highly upregulates COX-2 expression, mostly by local leucocytes; yet inhibition of COX-2 leads to suppression of polymorphonuclear leukocyte adhesion to lung tissue, leading to a paradoxical inflammatory outburst in later phases; therefore, the late anti-inflammatory effect of COX-2 is crucial to a timely resolution of acute lung injury (12).

Several studies have proved that COX-2 inhibition in a small-intestine IRI model alleviates tissue injury. The antioxidants resveratrol, curcumin and modafinil suppressed COX-2 expression in the small intestine following intestinal IRI (7, 18). In lung tissue, significant overproduction of COX-2 is seen after intestinal IRI, which is also reflected in accelerated apoptosis, angiogenesis and promoted lung fibrosis, ultimately leading to structural and functional lung injury (5). In our study we noticed gradual increases of COX-2 in the rat lung after intestinal IRI, with a significant (P-value < 0.001) and almost four-fold increase after 24 h of reperfusion. Betanin pre-treatment suppressed immunohistochemical COX-2 expression in lung tissue, in both early (P-value < 0.05) and late (P-value < 0.001) reperfusion periods, but it did not reduce the expression to the baseline level.

In a model of acid-induced lung injury, it was demonstrated that polymorphonucleocyte levels peaked within 12 h of insult, but lung macrophage and lymphocyte levels increased incrementally up to 48 or 72 h, and inhibition of the COX-2 pathway resulted in suppression of lung cytokine expression (9). We observed an upward trend in COX-2 expression in lung tissue after intestinal IRI only in rats denied betanin pre-treatment, while in the pre-treated animals during the reperfusion period a stable number of positive cells was noticed. From our previous results it is evident that IRI also altered COX-2 pathways in the rat small intestine, where primarily cells of inflammatory origin, including macrophages and mucosal mast cells, were positive for COX-2 (29).

This regulation of COX-2 and inflammatory cells in lung tissue has a representative microenvironment in the cellular changes in the bronchiolar epithelium, where club cells (CCs) are centrally involved. Detoxification of xenobiotics and oxidant gases, control of inflammation and maintenance of ciliated cell populations are the roles of CCs, the most frequent epithelial cells in bronchioles. Their role is still not fully understood, but it is known that they are a source of club cell secretory protein (CC16), which plays a role in mediating inflammation and has anti-inflammatory and immunosuppressive properties in physiological conditions (22). This protein modulates the production and activity of phospholipase A2, interferon-γ and TNF-α in the epithelial lining fluid. We observed strong cytoplasmic COX-2 positivity within cells of the epithelium, histologically suggesting mostly non-ciliated secretory CCs. Semiquantitative analysis revealed a gradual increase of COX-2^+^ cells within terminal bronchioles in group A rats peaking after 24 h of reperfusion. Quite a different pattern was noticed in betanin-treated rats, COX-2^+^ declining from its peak values after 1 h reperfusion and stabilising thereafter. The expression of COX-2 in CC may reflect changes in their secretory activities. In the CCs of the terminal bronchioles, COX-2 protein was present and was strongly induced 24 h after vanadium pentoxide exposure, so Bonner *et al*. (4) proposed that the inflammatory progression could be delayed by elicitation of prostaglandin E2 by COX-2.

Cell renewal is crucial in order to maintain metabolism and tissue vitality. As components of the replication and repair machinery, PCNAs play an essential role in nucleic acid metabolism (10). The initiation by intestinal IRI of cellular reparative/proliferative changes is well documented. Chen *et al*. (6) demonstrated that intestinal IR caused significantly higher PCNA expression at 2–12 h of reperfusion, indicating that the repair process was initiated by IR and continued afterwards. Similar results were observed in more studies, which concluded that intestinal IR significantly altered proliferation and repair within damaged tissue (1, 24). In our study we observed greater PCNA positivity with time in the groups given no betanin, peaking 24 h after reperfusion, with a more than two-fold increase from the level in the early reperfusion period. In contrast, in betanin-treated rats biopsied in the early reperfusion period (B1), lung tissue exhibited significantly higher PCNA expression than the non-betanin-treated rats and lower expression afterwards. The rise in PCNA positivity may related to exacerbation of the lung injury following intestinal IR, to which over-expression of COX-2 during reperfusion may also contribute. It is known that intestinal IRI has a negative impact on distant organs *via* an inflammation-driven pathway and increased apoptosis, and that it alters the histopathological structure of affected organs. However, less is known about the regeneration of distant organs following intestinal IRI. The findings of our study led to the conclusion that intestinal IR triggers nuclear PCNA expression in lung tissue after the insult. An upregulated pattern was observed in IR groups peaking after 24 h of reperfusion. In the betanin-pre-treated groups, this pattern was reversed. This may indicate that IR injuries induce the need for rapid reparative processes within affected organs through tissue changes and, more specifically, cellular changes.

Some limitations of our experimental study deserve to be mentioned. The application of a possibly protective substance prior to ischaemic insult does not reflect daily clinical practice where arterial occlusions are mainly acute; therefore, any method applied prior to vascular events is more beneficial in elective procedures. Secondly our study focuses entirely on the tissue and cellular impact of lung injury after IRI, thus not providing an insight into the biochemical or molecular level of the inflammatory process.

## Conclusion

The current experimental study provided evidence that intestinal IRI in a rat model drove rapid and early inflammatory changes in lung tissue, and that parenteral pre-treatment with betanin attenuated the acute injury to that tissue inflicted remotely by IR. Besides lessening the tissue injury at the histopathological level, betanin’s antioxidant capacity suppressed the COX-2-mediated inflammatory response in different populations of cells.
